# The post-reactive structures of *Leishmania major* UDP-sugar pyrophosphorylase provide insights into the product release mechanism

**DOI:** 10.1128/spectrum.00911-25

**Published:** 2025-10-10

**Authors:** Ohm Prakash, Jana Führing, Petra Baruch, Roman Fedorov, Françoise H. Routier

**Affiliations:** 1 Institute for Clinical Biochemistry, Hannover Medical School, Hannover, Germany; 2 Institute for Biophysical Chemistry/Research Division for Structural Biochemistry, Hannover Medical School, Hannover, Germany; Institut de recherche pour le developpement, Montpellier, France

**Keywords:** enzyme catalysis, pyrophosphorolysis, nucleotide sugar

## Abstract

Biosynthesis of the nucleotide sugars UDP-glucose (UDP-Glc) and UDP-galactose (UDP-Gal) is intimately connected and essential for the viability of trypanosomatid parasites. In the genus *Leishmania*, it is controlled by the UDP-glucose pyrophosphorylase (UGP) and UDP-sugar pyrophosphorylase (USP). In contrast to UGP, USP has a broad substrate specificity and may generate several UDP-sugars *in vitro,* including UDP-Glc and UDP-Gal. This enzyme, present in protozoan parasites (including *Leishmania* species and *Trypanosoma cruzi*) and in plants, most likely plays a role in salvaging monosaccharides. In order to gain a detailed mechanistic understanding of USPs, we determined high-resolution X-ray structures of *Leishmania major* USP (*Lm*USP) in post-reactive states. Several positions of the byproduct pyrophosphate (PP*i*) were identified and revealed a product release channel in the forward reaction, as well as the geometries of post-reactive Michaelis product complexes. The conformational changes of functional loops (hinge loop-1, hinge loop-2, and the nucleotide-binding loop) showed dynamic effects accompanying the product release process. Structural information about the post-reactive states of *Lm*USP also includes the metastable binding position of a magnesium (Mg^2+^) ion in the active site. The proposed product release mechanism was substantiated by molecular dynamics simulations and can serve as a model for other UDP-sugar pyrophosphorylases.

**IMPORTANCE:**

To survive in the hostile environment of the sandfly gut, the parasite *Leishmania* relies on a range of phosphoglycans made of mannose-phosphate and galactose. In these glucose-limiting conditions, mannogen potentially serves as a reservoir for the synthesis of these crucial glycoconjugates, whereas galactose likely arises from recycling. The enzyme UDP-sugar pyrophosphorylase (USP) is responsible for the activation of this monosaccharide. This enzyme has a relaxed specificity and converts UTP and a range of sugar-1-phosphate to the corresponding UDP-sugar and pyrophosphate (PP*i*). Here, we determined high-resolution X-ray structures of *Leishmania major* USP (*Lm*USP) in post-reactive states. The data provide insight into the product release mechanism for UDP-sugar pyrophosphorylases. Considering the conservation of the residues involved in the coordination of PP*i* amongst USP enzymes, this mechanism is relevant for all USPs. This work completes our knowledge of the catalytic mechanism of trypanosomatid uridylyltransferases, which are genetically validated drug targets.

## INTRODUCTION

Parasites of the genus *Leishmania* are responsible for a set of human diseases collectively called leishmaniases. These parasites are surrounded by a dense glycocalyx rich in carbohydrates that protects the parasites from hostile environments and enables interaction with their host. Sugar-1-phosphate nucleotidyltransferases are required for the activation of monosaccharides in the form of nucleotide sugars, which are substrates for polysaccharide and glycoconjugate biosynthesis. The proteins of this family catalyze bidirectional reactions and require a metal ion as a cofactor (1). They share a common Rossmann-like α/β/α sandwich fold of their catalytic domains and follow an ordered sequential bi-bi catalytic mechanism for the forward and reverse reactions (2–5). In the forward reaction, binding of the nucleoside triphosphate stabilizes the active site in an optimal conformation for accommodating the sugar-1-phosphate. Binding of this second substrate leads to the formation of the pre-reactive geometry for the catalytic reaction (6–9). After catalysis, inorganic pyrophosphate (PP*i*) dissociation precedes the nucleotide sugar release. The order of all steps is reverted in the pyrophosphorolysis of nucleotide sugars.

**Fig 1:**
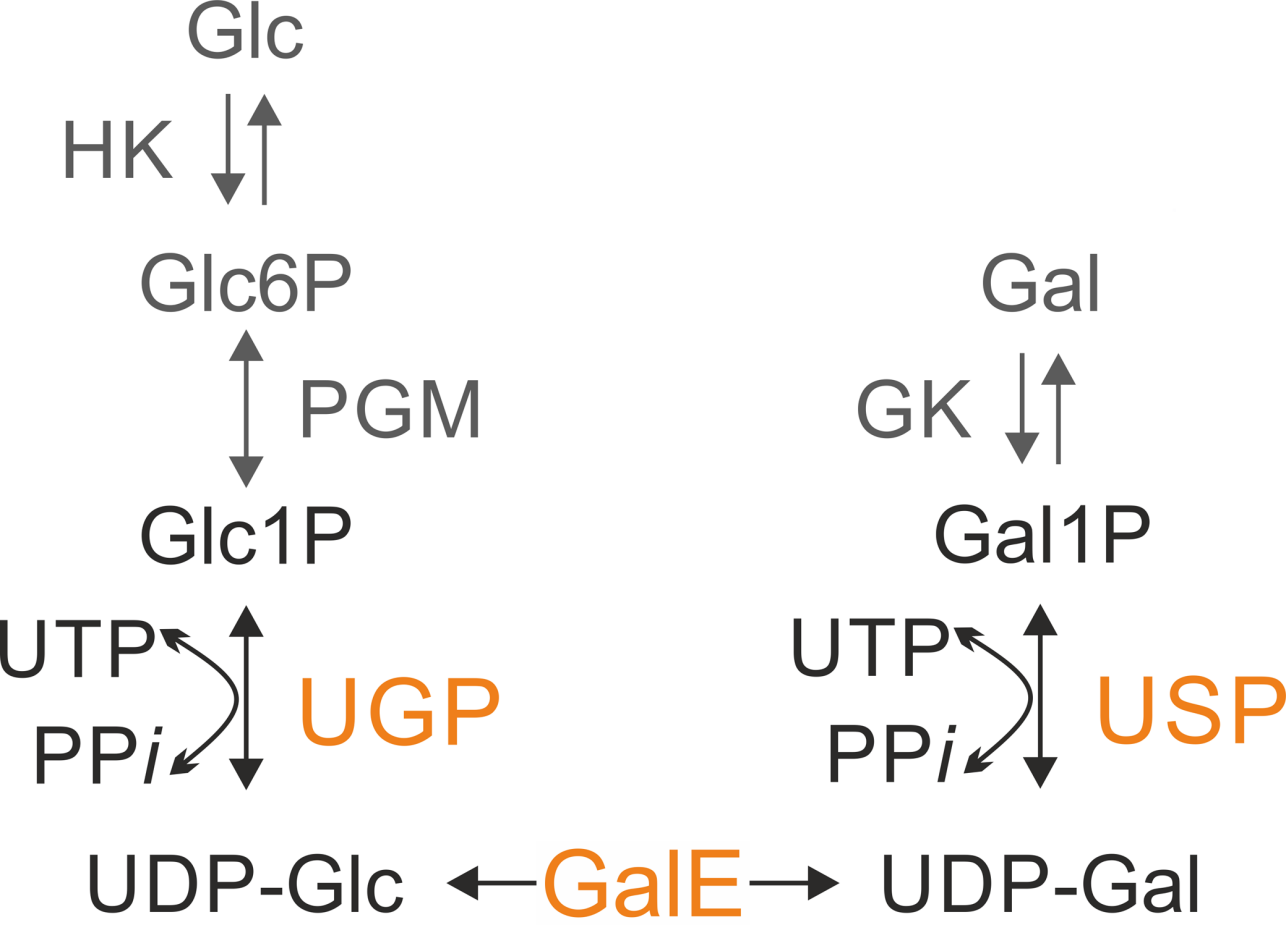
Biosynthesis of UDP-glucose (UDP-Glc) and UDP-galactose (UDP-Gal) in *Leishmania* parasites. UDP-glucose pyrophosphorylase (UGP; EC 2.7.7.9) synthesizes UDP-Glc by interconversion of glucose-1-phosphate (Glc-1-P) and uridine triphosphate (UTP) to inorganic pyrophosphate (PP*i*) and UDP-glucose (UDP-Glc). UDP-sugar pyrophosphorylase (USP; EC 2.7.7.64) activates several monosaccharide-1-phosphate, such as galactose-1-phosphate (Gal1P). The synthesis of UDP-Glc and UDP-Gal is connected by the UDP-glucose-4-epimerase (GALE). The nucleotide sugars UDP-Glc and UDP-Gal are precursors for a variety of glycosylation reactions and are essential for *Leishmania* survival. GK, galactokinase EC 2.7.1.6; HK, hexokinase EC 2.7.1.1; PGM, phosphoglucomutase EC 5.4.2.2.

The UDP-glucose pyrophosphorylase (UGP; also known as UTP glucose-1-phosphate uridylyltransferase; EC 2.7.7.9) and UDP-sugar pyrophosphorylase (USP; or UTP sugar-1-phosphate uridylyltransferase; EC 2.7.7.64) belong to the uridylyltransferase subfamily of the nucleotidyltransferase superfamily. UGP is a specific enzyme found in both prokaryotes and eukaryotes and is responsible for the *de novo* biosynthesis of the nucleotide sugar UDP-glucose (UDP-Glc) (10–12). It interconverts UTP and glucose-1-phosphate (Glc-1P) into UDP-Glc and inorganic pyrophosphate (PP*i*) (Fig. 1). In contrast to UGP, USP has only been described in protozoan parasites (including *Leishmania* species and *Trypanosoma cruzi*) and in plants but is absent from vertebrates (11, 13). It displays a broad substrate specificity and can activate a range of sugar-1-phosphates (sugar-1P) into the corresponding UDP-sugar (14). *In vivo*, USP most likely plays a role in salvaging sugars released during cell turnover (11, 13).

UDP-galactose (UDP-Gal) can be produced *de novo* from UDP-Glc by the action of the UDP-Glc 4-epimerase (GalE). The biosynthesis of UDP-Glc and UDP-Gal is thus intimately connected. (Fig. 1). These two nucleotide sugars play a central role in the formation of the cell glycocalyx, in DNA glucosylation, as well as in the glycoprotein quality control and are essential for *Leishmania* viability (15). Conditional destabilization of USP in UGP-deficient parasites leads to growth cessation, showing the importance of these enzymes (15). Similarly, these nucleotide sugars and the enzymes synthesizing them are essential for the survival of the trypanosomatids *Trypanosoma brucei* and *T. cruzi* (16–18). The parasite *Leishmania major* is also able to take up galactose from the environment and incorporate it into its glycoconjugates. In this salvage pathway, galactose is first phosphorylated by the galactokinase (19, 20), and the resulting galactose-1P (Gal-1P) is activated to UDP-Gal by USP (Fig. 1) (13, 21). In the absence of glucose, such as in the gut of the insect vector, salvage of galactose via USP would enable the synthesis of UDP-Gal (20) and sustain the synthesis of galactose-rich phosphoglycans. These are essential for survival of *Leishmania* in the insect gut and infection of mammals (22). The previously obtained structures of USP and UGP kinetic states (Table S1) provided substantial information about the molecular mechanism and conformational changes during the forward reaction (6, 7, 9). The overall structure of these enzymes can be divided into the N-terminal, catalytic middle (M), and C-terminal domains (Fig. S1). The M-domain includes the nucleotide-binding (NB) and sugar-binding (SB) loops, which play essential roles in substrate binding.

Important structural changes occur at the NB-loop during the formation of the UTP-bound state from the substrate-free enzyme. These changes not only increase the affinity of the active site for UTP but also propagate toward the SB-loop area and enable the binding of the sugar-1P. The latter effect is facilitated by the allosteric interaction networks involving the highly conserved central β-sheet of the M-domain. The binding of sugar-1P triggers domain rearrangements of large magnitude that lead to the closing of the active site and lock the structure in a closed conformation optimal for catalysis (9, 23). Both USP and UGP utilize this mechanism for the stabilization of the sugar-1P during catalysis (7, 9).

After the product formation, the locking process is reversed to facilitate product release and complete the enzymatic cycle by returning the protein to the substrate-free state, ready for the next round of biosynthesis. The high-resolution post-reactive state structure of *L. major* UGP (*Lm*UGP) provided evidence for the unlocking mechanism of the enzyme after catalysis and subsequent product release (9). Dissociation of PP*i* and Mg*^2+^* causes destabilization of the NB-loop, which propagates towards the SB-loop and phosphate moiety of UDP-sugar via a seven-stranded β-sheet. The resulting destabilization of both the NB-loop and the SB-loop areas activates the enzyme for UDP-Glc dissociation and the closed-to-open state structural transition of *Lm*UGP. Currently, there is no experimental structural information describing the intermediate states associated with product release and its mechanism in USPs. In this study, we obtained experimental structural evidence for the conformational changes in *Lm*USP during the initial product release steps and identified the existence of a PP*i* exit channel in this enzyme. The new data provide insights into the release mechanism of activated sugars—the essential metabolites for the viability of trypanosomatid parasites.

## RESULTS

### The post-reactive state structure of *Lm*USP

**Fig 2:**
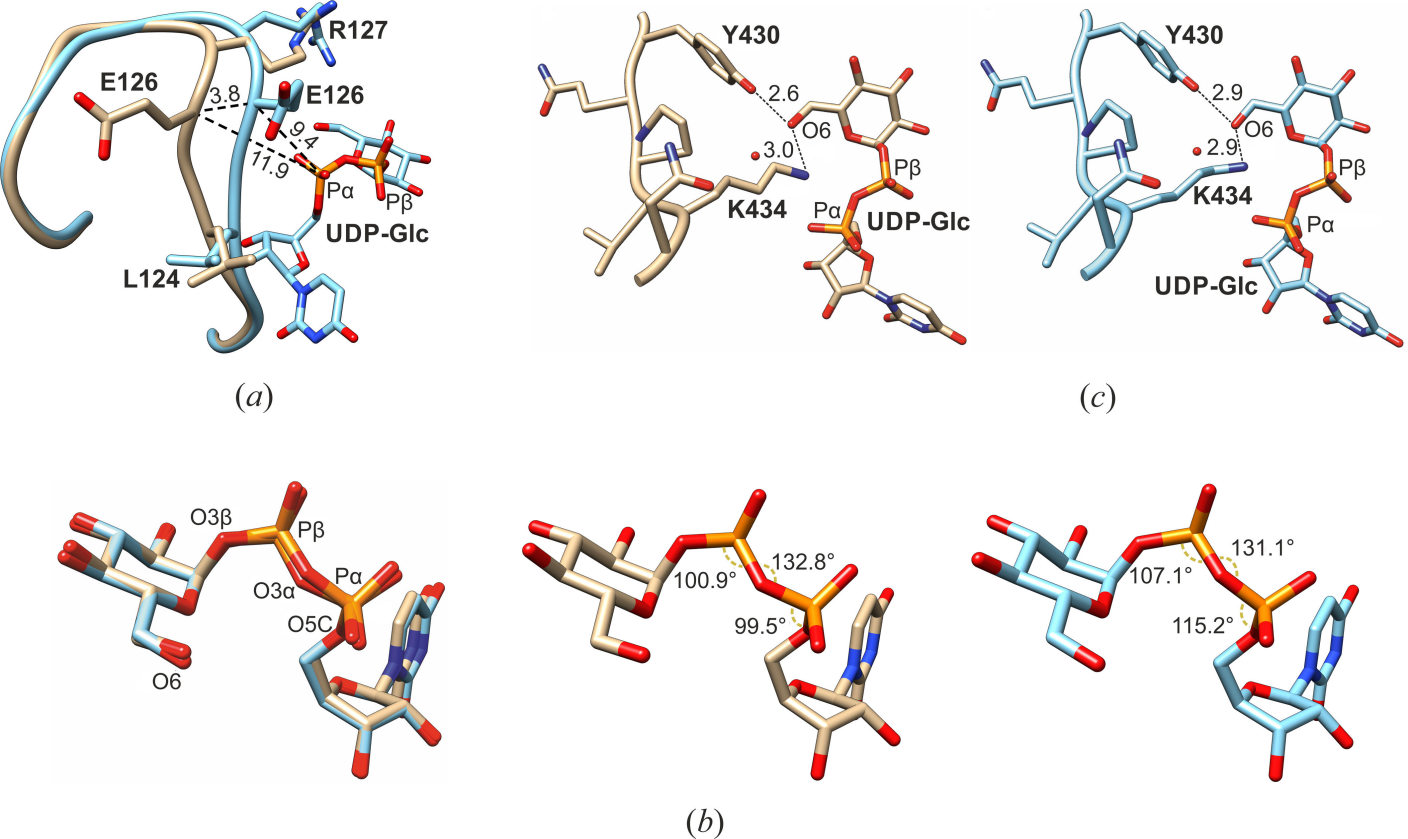
Comparison of the post-reactive *Lm*USP:UDP-Glc:PP*i* complex (in beige) and *Lm*USP:UDP-Glc complex (PDB code: 3OH4 in cyan). (**a**) Superposition of the two complexes showing the shift of the NB-loop. The distances in Å of shifted residue E126 from the α-phosphate moiety of UDP-Glc are indicated. (**b**) Superposition of UDP-Glc in the active site showing the repositioning of the O6 atom at the glucose moiety. The O3β-Pβ-O3α, Pβ-O3α-Pα, and O5C-Pα-O3α angles of UDP-Glc are indicated. (**c**) UDP-Glc:O6 distance from K434 and Y430.

To study the post-reactive state structure of *Lm*USP, we co-crystallized the enzyme with UDP-Glc and soaked the obtained crystals in a cryo-protecting solution containing PP*i* before freezing the samples in liquid nitrogen for data collection. The structure was solved by molecular replacement and refined to 2.4 Å resolution. The final *2F_o_-F_c_* electron density had excellent overall quality, and the omit density map allowed the unbiased and unambiguous fitting of the products UDP-Glc and PP*i* positions 1 and 2 with the real-space *R*-factor values of 0.07, 0.14, and 0.16, respectively (Fig. S2a and b). The only areas where weak electron density was observed were the loops α7–α8 (residues 237–250) and α10–β14 (residues 339–350). Due to their inherent flexibility, these regions have not been resolved in the previously published structures of *Lm*USP in the apo-form (PDB code: 3OGZ) and in complex with UTP (3OH0), UDP-Ara (3OH3), UDP-Glc (3OH4), UDP-Gal (3OH2), and UDP-GalA (3OH1) (Fig. S1c). To resolve these regions, we improved the electron density by the density modification procedure (DM in the CCP4 software suite) using Hendrickson-Lattman coefficients resulting from the omit map calculations. This procedure helped increase the signal-to-noise ratio in the electron density for the loops α7–α8 and α10–β14, which allowed building the structural model for the missing residues and thus completing the *Lm*USP structure. The overall conformation of the enzyme was similar to the UDP-Glc bound state, but substantial changes were observed in the NB-loop region (Fig. S1c). A 110° angle flip of the side chain of E126 and distortions of L124 and R127 residues cause the NB-loop to shift away from the phosphate moiety of UDP-Glc (Fig. 2a). As a result, the NB-loop acquires an intermediate conformation between the closed-bound and the UTP-bound states with a semi-open active site cleft.

### UDP-Glc has a constricted geometry in the post-reactive state

The nucleotide moiety of UDP-Glc in the post-reactive *Lm*USP:UDP-Glc:PP*i* state (Fig. 2b) has a strained intermediate geometry between the pre-reactive *Lm*USP:UTP complex and the *Lm*USP:UDP-Glc complex (Fig. 2b) that is formed after the dissociation of PP*i* (PDB codes: 3OH0 and 3OH4, respectively). The phosphate moiety in the post-reactive state differs from the UDP-Glc state in the O5C-Pα-O3α and O3β-Pβ-O3α angles, which are decreased from 115.2° to 103.3° and from 107.1° to 104.9°, respectively (Fig. 2b). The comparison further revealed that the distances between the O3α atoms, the O1α atoms, and the bases in UDP-Glc in the *Lm*USP:UDP-Glc and *Lm*USP:UDP-Glc:PP*i* complex structures differ by 0.4 Å, which exceeds the coordinate error of 0.3 Å for these structures (Table 1).

**TABLE 1:** Crystallographic data and refinement statistics

Protein/complex	*Lm*USP:UDP-Glc:PP*i*	*Lm*USP:UDP-Glc:VO_4_:Mg^2+^
PDB code	8TG2	8TGS
Group	*C*2	*C*2
Cell parameters: *a*, *b*, *c* [Å]	107.1, 121.9, 61.0	108.1, 122.4, 61.2
*α*, *β*, *γ* [°]	90, 105.7, 90	90, 105.6, 90
Beamline^a^	P13/PETRA-III	P13/PETRA-III
Wavelength: [Å]	0.9763	0.9763
Resolution limit: (high-resolution shell)^b^ [Å]	78.7–2.4 (2.5–2.4)	79.3–2.2 (2.3–2.2)
No. of observations/unique reflections	203,829/29,455	268,327/38,888
Completeness: total/high [%]	100/99.9	99.9/99.8
Redundancy: total/high	6.9/6.7	6.9/7.0
<*I*/*σ*(*I*)>: total/high	19.0/2.4	15.4/2.2
*R*_sigma_: total/high [%]^c^	2.7/40.2	3.4/42.8
*R*_int_: total/high [%]^c^	4.5/51.7	4.8/59.2
CC(1/2): total/high [%]	99.9/90.1	99.9/80.7
Wilson B-factor: [Å^2^]	76.04	70.6
Crystal mosaicity: [°]	0.06	0.11
Coordinate error (Å)^d^	0.32	0.33
Included amino acids	3–610	3–610
No. of protein atoms	4,706	4,706
No. of waters	209	244
Ions	UDP-Glc, 2 PPi	UDP-Glc, VO_4_, Mg^2+^
*R*_work_/*R*_free_: [%]^e^	20.6/23.6	21.1/24.2
r.m.s.d.: bonds [Å]/angles [°]	0.009/1.2	0.008/1.3
Mean B-factor: [Å^2^]		
Overall/protein/ligands/solvent	71.3/77.4/75.2/72.2	68.7/74.6/62.6/69.5

^a^ Reference (24).

^b^ High-resolution cutoffs were applied to resolution shells that had the average error in the intensities *R*_sigma_ > 50% or had sustained high radiation damage.

^c^ *R*_sigma_ = ∑[*σ*(*F*_o_^2^)]/∑[*F*_o_^2^]; *R_int_* = ∑|*F*_o_^2^ − *F*_o_^2^(mean)|/∑[*F*_o_^2^].

^d^ Based on Luzzati plot.

^e^ *R*_free_ is calculated for a randomly chosen 5% subset of reflections.

The position of the O6 atom of Glc is slightly shifted away from the highly conserved K434 toward Y430 in the post-reactive state (Fig. 2c). In the substrate-free and UTP-bound kinetic states, the amino group of the K434 side chain forms H-bond interactions with the hydroxyl group of Y430 (7). During sugar-1P binding, this interaction is lost as the K434 amino group moves 4.5 Å away and acts as the counter ion for assisting the S_N_2 catalytic reaction (7). The movement of the Glc O6 depicted in Fig. 2c might be accompanied by a shift of the K434 side chain toward the vicinity of Y430, ideal for H-bonding. This could disrupt the interactions of Y430 and K434 with the glucose moiety, thereby weakening its binding to the catalytic site and inducing the dissociation process.

### The PP*i* binding sites reveal a series of post-reactive Michaelis product complexes

**Fig 3:**
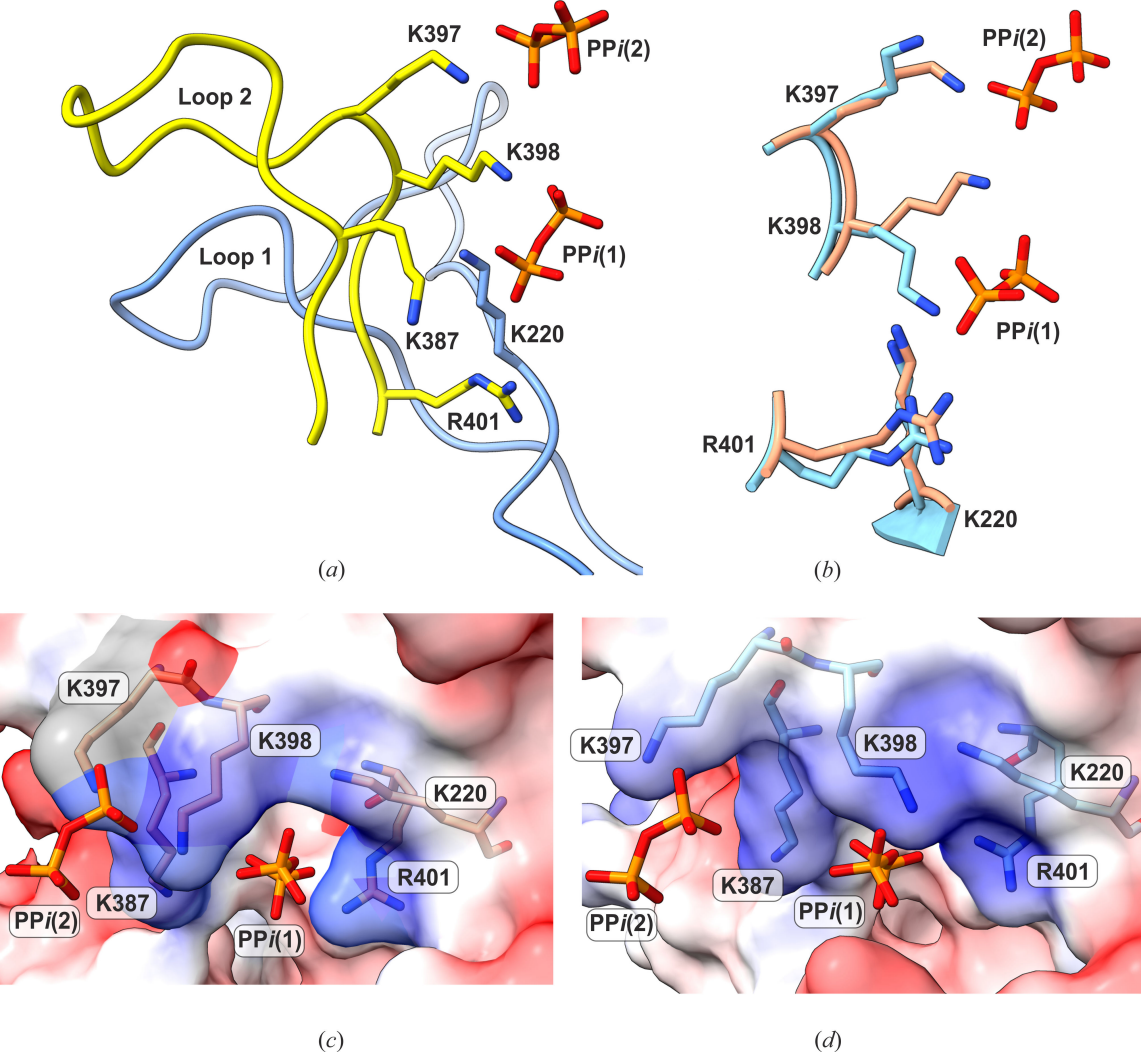
PP*i* binding locations in *Lm*USP. (**a**) The newly identified PP*i* binding sites (1) and (2) on *Lm*USP. (**b**) K398 orientation in *Lm*USP:UDP-Glc:PP*i* complex (beige) compared to the one in the *Lm*USP:UDP-Glc structure (cyan; PDB code: 3OH4). (**c**) Electrostatic surface potential of the binding groove (blue: positive potential; red: negative potential). (**d**) The superposition of PP*i* binding sites on the molecular surface of the *Lm*USP:UDP-Glc structure (PDB code: 3OH4) demonstrates the role of K398 as a molecular gateway for PP*i* access.

The crystal structure of the *Lm*USP:UDP-Glc:PP*i* complex determined in this work revealed two positions of the pyrophosphate in the active site cleft (Fig. 3). The first position—PP*i* (1)—is directly adjacent to the UDP-Glc binding site and represents the first stable binding position after the catalytic reaction. Comparison of the crystal structure with the *Lm*USP:UDP-Glc state revealed additional conformational changes at the two long loops (loop 1 and loop 2), which emanate from the interface of the N-terminal domain and M-domain. These so-called hinge loops are involved in the lock mechanism of *Lm*USP and are seen to move over the active site cleft during enzymatic catalysis (7). In the *Lm*USP:UDP-Glc:PP*i* structure, these loops are involved in the binding of PP*i*(1) (Fig. 3a) in a positively charged binding groove surrounded by K387, K398, R401, and K220 (Fig. 3a and c). The side chain of K398 is flipped by 112° (Fig. 3b) compared to the *Lm*USP:UDP-Glc structure, which creates an access channel toward the groove (Fig. 3c and d). The binding of PP*i* (1) between K398 and E126 leads to the reorientation of the K398 side chain to form a hydrogen bond with PP*i*(1) (Fig. 3b) and the shift of E126 to accommodate the ligand (Fig. 2a). These effects are responsible for a major shift of the NB-loop described in “The post-reactive state structure of *Lm*USP,” above.

The PP*i* (2) molecule is bound further out toward the *Lm*USP surface. Its position is stabilized by the interactions with the loop 2 residues K397 and K398. The PP*i* (2) is also bound near the loop 2 at the outermost part of the active site cleft. Due to this location and substantial solvent exposure, it is likely that the PP*i* (2) corresponds to a pre-dissociation state of the first *Lm*USP product. In both the PP*i* (1) and PP*i* (2) structures, no Mg^2+^ ion could be observed, which indicates the higher lability of the metal ion and the possibility that it may dissociate first.

Thus, the described positions of PP*i* reveal the product release channel in the forward reaction as well as the structures of the post-reactive Michaelis product complexes. The conformational changes of loop 1, loop 2, and the NB-loop indicate that these effects of protein dynamics are essential for the product dissociation process. This information can also be interpreted as the initial stages of the UDP-Glc pyrophosphorolysis, which is the reverse reaction of *Lm*USP.

### Mg^2+^ accompanied PP*i* release/entry

**Fig 4:**
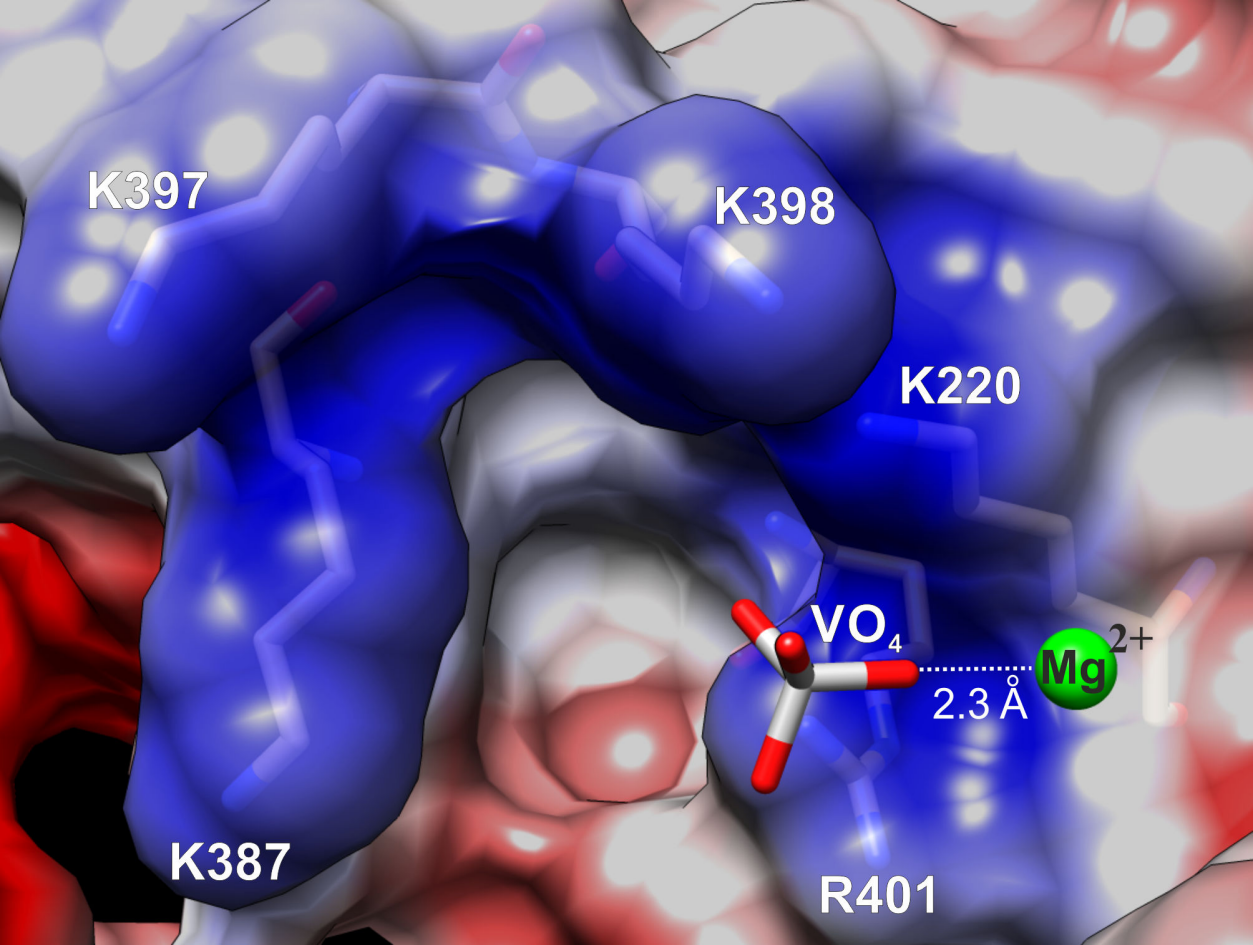
Metastable position of a Mg^2+^ ion in the active site of *Lm*USP coordinated by the VO_4_ analog of phosphate.

In an alternative approach to trap the post-reactive kinetic Michaelis states of *Lm*USP, we co-crystallized *Lm*USP with UDP-Glc in the presence of magnesium ions and soaked the crystal in a cryo-solution containing orthovanadate (VO_4_) as a non-reactive analog of phosphate (25). The structure, solved by molecular replacement and refined to 2.2 Å resolution, showed good electron density for UDP-Glc and VO_4_ (Fig. S2c and d). The flexible loops α7–α8 and α10–β14 were resolved using the same procedure as described in “The post-reactive state structure of *Lm*USP,” above. The orthovanadate was bound at the same location as PP*i* (1). The analysis of shape and stereochemical features of the electron density at this site revealed the specific geometry, consistent with a Mg²^+^ ion coordinated by VO_4_ (Fig. 4). The conformational changes in the hinge loops and the NB-loop areas observed for the *Lm*USP:UDP-Glc:PP*i* complex structure were also observed in the *Lm*USP:UDP-Glc:VO_4_:Mg^2+^ complex. These include the active site cleft widening and the side chain flips for E126 and K398 residues.

Thus, the *Lm*USP:UDP-Glc:VO_4_:Mg^2+^ structure confirmed the effects observed in the *Lm*USP:UDP-Glc:PP*i* complex and revealed the post-reactive Mg^2+^ position in the active site of *Lm*USP. The Mg^2+^ binding site represents the intermediate state for magnesium coordination during the enzymatic cycle.

### Positively charged residues guide PP*i* release/entry

**Fig 5:**
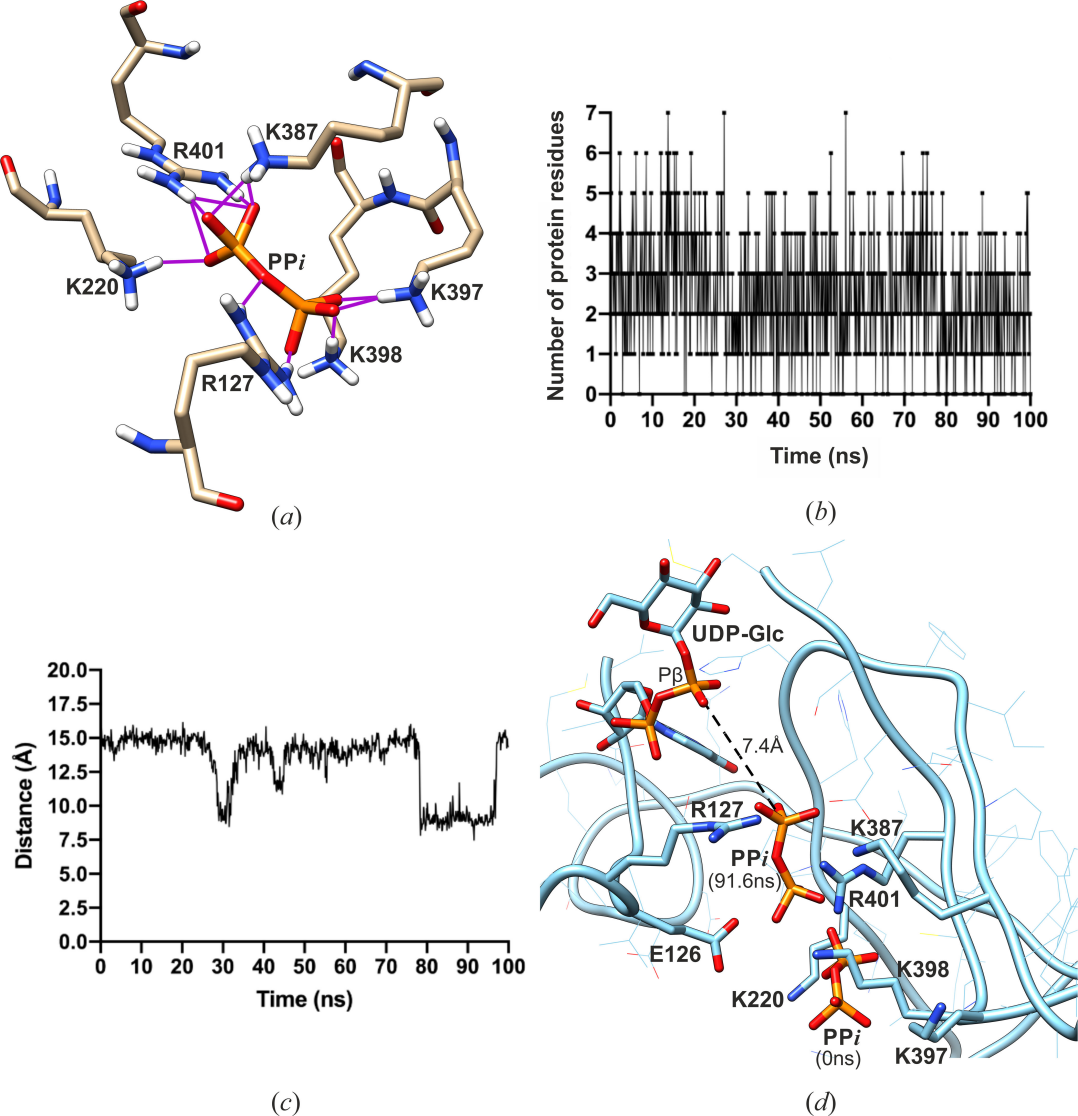
Product release pathway studied by molecular dynamic simulation. (**a**) Representative image of the H-bonds formed between PP*i* and the positively charged residues. The H-bonds are shown as magenta lines. Image generated at the time point of 13.7 ns of the MD trajectory. (**b**) Number of protein residues involved in H-bonds with PP*i* along the MD trajectory. (**c**) Distance between PP*i*:P1 atom and UDP-Glc:Pβ atom measured at various time points of the simulation. (**d**) The shortest distance of 7.4 Å between PP*i*:P1 atom and UDP-Glc:Pβ atom near the glucose moiety at 91.6 ns. PP*i* at 0 ns and at 91.6 ns of simulation time is shown in the line and the ball-and-stick representations, respectively. UDP-Glc is shown in the ball-and-stick representation, and the distance is shown as dashed black lines.

Using the position of PP*i* (1) in the *Lm*USP:UDP-Glc:PP*i* complex structure as a starting point, a molecular dynamics (MD) simulation was performed to study the product release pathway of PP*i*. During the 100 ns of the MD trajectory, the PP*i* product moved back and forth between the UDP-Glc binding site and the exit site near the protein surface (Movie S1). Analysis of the 1,000 frames generated during the 100 ns simulation run unveiled an active role of positively charged residues in loop 1 and loop 2 in facilitating the movement of PP*i* between the catalytic center and the protein surface. The MD simulation shows that R127, K220, K387, K397, K398, and R401 (which are conserved among USPs, see Fig. S5) are actively involved in the dynamic H-bond and electrostatic interactions with PP*i* (Fig. 5a and b). These interactions facilitate the reorientation of PP*i* and its movement between the UDP-Glc and the exit sites. The movement of PP*i* toward (or away from) UDP-Glc was monitored by the distance between the PP*i*:P1 atom and UDP-Glc:Pβ atom at several time points (Fig. 5c). This distance along the dynamic trajectory shows how PP*i* reaches the stable, energetically favorable position at the time point of 78.3–96.4 ns after testing several metastable local minima (e.g., at 30.3–31.5 ns and at 43.3–44.6; Fig. 5c). The shortest measured distance of 7.4 Å between the PP*i*:P1 atom and UDP-Glc:Pβ atom occurred at the 91.6 ns time point (Fig. 5d). This geometry is similar to the one observed in the experimental crystal structure of the post-reactive state complex of *Lm*UGP (PDB code: 4M2A, Fig. S3). Residues E126 and E403 also influence the movement of PP*i* indirectly via intramolecular H-bonds with the positively charged residues.

## DISCUSSION

**Fig 6:**
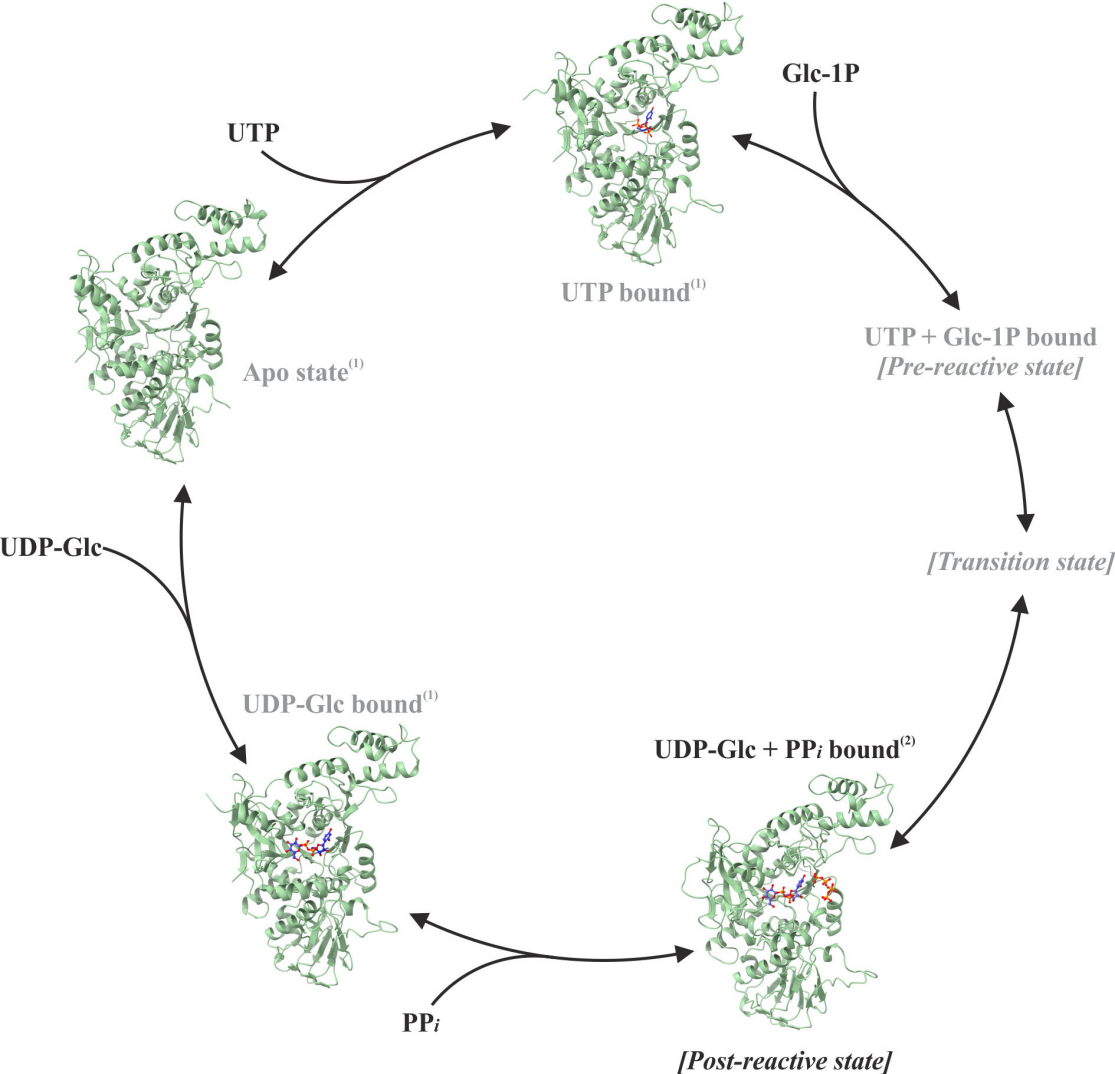
Reaction cycle of *Lm*USP. Previously known structures (1, reference 7) and non-crystallizable states are labeled in gray and new structures (2, this work) in black. PDB codes: Apo state 3OGZ, UTP bound state 3OH0, UDP-Glc + PP*i* bound 8TG2, UDP-Glc bound 3OH4.

The structure of *Lm*USP presented here in its post-reactive kinetic state closes a previously existing gap in the *Lm*USP reaction cycle (Fig. 6) and provides the first structural evidence for the product release mechanism in the uridylyltransferase subfamily of nucleotidyltransferases. This mechanism directly controls the release of activated sugars in *L. major*, which are essential for the viability of these parasites (15). The reaction cycle of *Lm*USP begins with the binding of UTP to a substrate-free (apo) protein whose structure has an open conformation (6, 13). UTP binding triggers conformational changes, mainly at the NB-loop and the SB-loop regions, which leads to the formation of a complementary geometry and charge distribution for binding the second substrate, sugar-1P. The binding of the sugar-1P is accompanied by large-scale conformational changes, which involve both global movements of the protein domains and local rearrangements of the residues. Stabilization of the second substrate locks the enzyme in a closed state, optimal for catalysis (7, 9). Upon completion of the reaction, the release of PP*i* and Mg^2+^ initiates the unlocking, which, in turn, activates the enzyme for UDP-sugar release and the closed-to-open conformational transition. The stable kinetic states of the enzyme during its open-to-closed transition were previously described (7). However, no structural information was available to reveal the conformational changes associated with the sequential release of products after the catalytic reaction step (Table S2). The structural changes described here during the PP*i* release are assumed to occur in reverse order during the PP*i* entry for UDP-sugar pyrophosphorolysis (the reverse reaction).

The critical roles played by the NB-loop in nucleotide binding and in the activating of the SB-loop area for the reaction have been previously described (9). The NB-loop also plays an essential role in the product release process. The comparison of *Lm*USP:UTP (PDB code: 3OH0) and *Lm*USP:UDP-Glc (PDB code: 3OH4) (7) complex structures with the structure of the *Lm*USP:UDP-Glc:PP*i* complex determined in this study revealed that R127 and K134 are likely to coordinate the leaving PP*i* group immediately after catalysis (Fig. S4). This first coordination site is shielded from the solvent by the NB-loop upon conformational changes from the UTP-bound state to the closed state of the enzyme. In the post-reactive *Lm*USP structure, this site is no longer shielded due to the partial opening of the NB-loop, which creates an exit channel for PP*i* release (Fig. 2a). This is in agreement with the previously described unlocking mechanism via destabilization of the NB-loop and subsequent release of PP*i* with the Mg^2+^ ion (9).

The release of the UDP-sugar product is coupled with the process of the enzyme opening. The sugar moiety of the UDP-sugar is thought to dissociate first (9). Our structural data show the UDP-Glc to be in a highly constricted geometry, especially at the phosphate moiety in comparison with the previously described *Lm*USP:UDP-Glc complex structure (Fig. 2). The O6 atom in the glucose moiety is shifted away from the residue K434, which acts as a counter ion for the two negatively charged phosphates during the nucleophilic attack. The increased conformational strain around UDP-Glc and the opening of the active site cleft weaken the binding of UDP-Glc to the active site and eventually lead to product release.

During the dissociation process, the PP*i* ion binds at the interface between the M-domain and the N-terminal domain in a groove formed by positively charged residues K220, K397, K398, K387, and R401 from the hinge loop 1 and loop 2. The side chain of K398 flips by 112°, thus acting as a molecular gateway for PP*i* access (Fig. 3). The MD simulation study using the *Lm*USP:UDP-Glc:PP*i* complex structure and PP*i* (1) serving as an initial position of PP*i* demonstrated the importance of these positively charged residues in facilitating the movement of PP*i* between the catalytic center and the molecular surface. The results of the MD simulation support the functional role of the PP*i* access/release channel and its positively charged residues in *Lm*USP (Fig. 5). The second remote position of the PP*i* molecule, coordinated by the residues K397 and K398, is located at the outer edge of the product release channel. This likely represents the pre-dissociation state for PP*i* before it leaves the protein molecule.

Thus, both PP*i* positions reveal a series of post-reactive Michaelis product complexes. The PP*i* release mechanism involves the conformational changes of loop 1, loop 2, and the NB-loop, which likely facilitate the product release and direct PP*i* into the product release channel with the help of several positively charged residues. Interestingly, while the majority of active site residues are conserved between *Lm*USP and human (*Homo sapiens, Hs*)UGP, this is not the case for the six positively charged *Lm*USP residues involved in PP*i* exit (Fig. S6). This structural and mechanistic difference may be exploitable for development of selective allosteric *Lm*USP inhibitors.

The metastable position of the Mg^2+^ ion bound to the PP*i* analog VO_4_ in the *Lm*USP:UDP-Glc:VO_4_:Mg^2+^ complex structure provides additional information about the metal cofactor properties in the active site of *Lm*USP. Some nucleotidyltransferases, such as the innate immune sensors OAS1 and cGAS, *Mycobacterium tuberculosis* UDP-N-acetylglucosamine pyrophosphorylase GlmU^MtB^ (26–29), or UGP from *Corynebacterium glutamicum* (*Cg*UGP) (30), use two Mg^2+^ ions for mechanisms of catalysis and exchange of reaction components. Others, such as *L. major* UGP or *Aspergillus fumigatus* UDP-GlcNAc pyrophosphorylase, require only one magnesium ion (9, 31). In this case, the positively charged side chain of a lysine residue occupies the position of the second Mg^2+^ ion (9, 31). In *Lm*USP, a conserved lysine residue (K434) is positioned in the close vicinity of the α-phosphate of UTP and the phosphate of the sugar-1P, suggesting that *Lm*USP also requires a single catalytic magnesium (7). This interpretation is supported by the observed electron density consistent with a single metal atom in the *Lm*USP:UDP-Glc:VO₄:Mg²^+^ complex described in this study.

This observation that Mg²^+^ can dissociate from the active site of *Lm*USP together with pyrophosphate is in agreement with a mechanism reported for the closely related *Lm*UGP (9). Meanwhile, structural data from other USP-related enzymes—in particular, bacterial UGPs in which Mg^2+^ binds phosphate moieties of nucleotide sugars (30, 32, 33)—illustrate the existence of alternative pathways for Mg^2+^ dissociation.

To summarize, the experimental structural data presented in this paper reveal the post-reactive kinetic states (or pre-reactive reverse reaction kinetic states) in the enzymatic cycle of *Lm*USP. The identification of a positively charged PP*i* access/release channel and the description of its conformational properties provide the first experimental insight into the details of the product release mechanism for UDP-sugar pyrophosphorylases. The MD simulations support our interpretation of the product release mechanism and provide a continuous model of this process. Considering the conservation of the residues involved in the coordination of PP*i* amongst USP enzymes (Fig. S5), this mechanism is most likely applicable to all USPs. The PP*i* access/release channel description is an essential, previously unknown part of the catalytic mechanism of trypanosomatid uridylyltransferases.

## MATERIALS AND METHODS

### Protein preparation and crystallization

Wild-type *Lm*USP was prepared as described by Damerow et al. (13). Crystallization was performed at 20°C using the vapor diffusion method. *Lm*USP was crystallized at a concentration of 22 mg/mL with 5 mM UDP-Glc and 10 mM dithiothreitol (DTT). The reservoir solution contained 200 mM ammonium acetate, 100 mM tri-sodium citrate, pH 5.4–5.6, and 15–17% polyethylene glycol-4000 (PEG-4000). Before flash cooling, the crystals were soaked in a reservoir solution supplemented with 5 mM PP*i* or VO_4_ and 15% ethylene glycol for cryoprotection.

### Diffraction data collection and structure determination

Diffraction data were collected at the EMBL Beamline MX1-P13, PETRA-III/DESY synchrotron, Hamburg, Germany, using PILATUS 6M (Dectris) detector (see Table 1 for details). The data sets were reduced with the XDS (34) program package. The structures of *Lm*USP:UDP-Glc:PP*i* complex and *Lm*USP:UDP-Glc:VO_4_:Mg^2+^ complex were solved by molecular replacement (CCP4 software suite [35]) using the protein part of the *Lm*USP :UDP-Glc complex structure (PDB code: 3OH4 [7]) as an initial model. The electron density calculation, model fitting, and refinement were continued with ARP/wARP (36), DM (37), COOT (38), REFMAC (39), and PHENIX (40) program packages and included both coordinate and individual B-factor refinement. During cyclic rounds of refinement and manual rebuilding using the COOT (38) program, ligands and solvent molecules were included in the models. The final models displayed good stereochemistry (see Table 1). The structure comparisons were performed using align and superposition commands in COOT and PyMol (https://www.schrodinger.com/).

### MD simulation

MD simulation was performed on the *Lm*USP:UDP-Glc:PP*i* complex structure using the NAMD Scalable Molecular Dynamics program (41) with the AMBER force field (42) and the TIP3P water model (43) with periodic boundary conditions. The model included the *Lm*USP protein with the products UDP-Glc and PP*i*, the counterions for the charged groups, and the explicit water environment. The active site, the NB-loop, and the two long loops forming the highly positively charged groove assumed to coordinate PP*i* were the active parts of the simulation. The positions of Cα atoms for the remote parts of the protein structure were restrained to maintain the experimentally observed protein fold. After the initial energy minimization, heating, and cooling cycles, as well as an equilibration of the system at constant pressure (1 atm), a 100 ns production run was carried out under the periodic boundary conditions with constant temperature (310 K). All molecular images of the simulation results were rendered in VMD (44) and UCSF Chimera 1.14 (45).

## Data Availability

Coordinates for the structures of *Lm*USP:UDP-Glc:PP*i* and *Lm*USP:UDP-Glc:VO_4_:Mg^2+^ complexes have been deposited to the Protein Data Bank with the accession codes 8TG2 and 8TGS, respectively.

## SUPPLEMENTAL MATERIAL

Supplemental figures and tables spectrum.00911-25-s0001.pdf Figures S1 to S6, and Tables S1 and S2.

Movie S1 spectrum.00911-25-s0002.avi Movement of the pyrophosphate product.

ASM does not own the copyrights to Supplemental Material that may be linked to, or accessed through, an article. The authors have granted ASM a non-exclusive, world-wide license to publish the Supplemental Material files. Please contact the corresponding author directly for reuse.
