# Supplementary material for: The post-reactive structures of Leishmania major UDP-sugar pyrophosphorylase provide insights into the product release mechanism
Source: Microbiol Spectr. 2025 Oct 10;13(11):e00911-25. doi: 10.1128/spectrum.00911-25 (PMC12584669; doi:10.1128/spectrum.00911-25)
Supplement: Supplemental figures and tables — Figures S1 to S6, and Tables S1 and S2. [file spectrum.00911-25-s0001.pdf]

The post-reactive structures of *Leishmania major* UDP-sugar pyrophosphorylase provide insights into the product release mechanism

- Supplementary figures and tables -

Ohm Prakash[a§], Jana Führing[a], Petra Baruch[b], Roman Fedorov[b1]* and Françoise H. Routier[a1]*

[a]Institute for Clinical Biochemistry, Hannover Medical School, Carl-Neuberg-Strasse 1, Hannover, 30625, Germany

[b]Institute for Biophysical Chemistry / Research Division for Structural Biochemistry, Hannover Medical School, Carl-Neuberg-Strasse 1, Hannover, 30625, Germany

[§]Current address: Protein-Protein Interaction Laboratory, The Francis Crick Institute, London, United Kingdom

[*]Correspondence: Routier.Francoise@mh-hannover.de; Fedorov.Roman@mh-hannover.de

[1] These authors contributed equally.

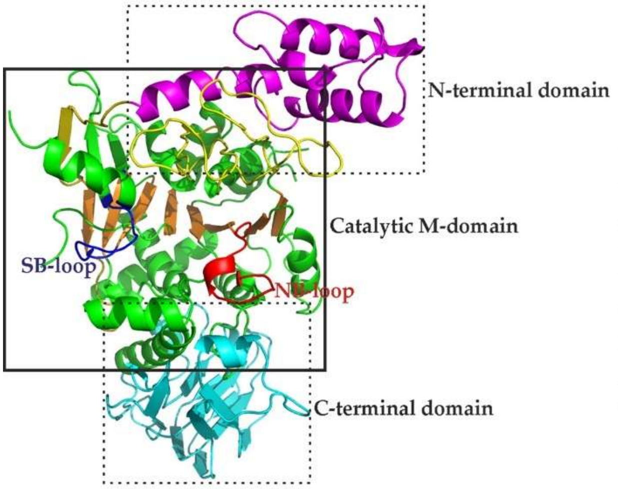
N-terminal domain
Catalytic M-domain
SB-loop
NB-loop
C-terminal domain

(*a*)

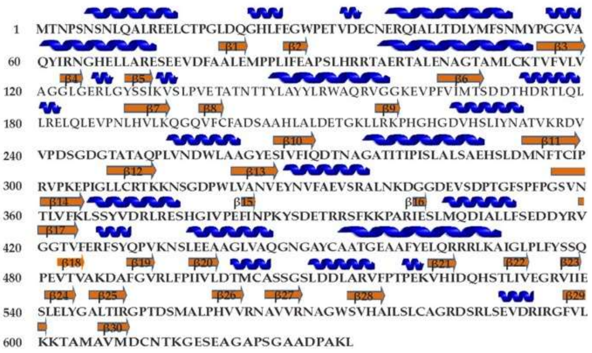
1 MTNPSNSNLQALREELCTPGLDQGHLFEGWPETVDECNERQIALLTDLYMFSNMYPGGVA
60 QYIRNGHELLARESEEVDFAALEMPPLIFEAPSLHRRTAERTALENAGTAMLCKTVFVLV
120 AGGLGERLGYSSIKVSLPVETATNTTYLAYYLRWAQRVGGKEVPFVIMTSDDTHDRTLQL
180 LRELQLEVPNLHVLKQGQVFCFADSAAHLALDETGKLLRKPHGHGDVHSLIYNATVKRDV
240 VPDSGDGTATAQPLVNDWLAAGYESIVFIQDTNAGATITIPISLALSAEHSLDMNFTCIP
300 RVPKEPIGLLCRTKKNSGDPWLVANVEYNVFAEVSRALNKDGGDEVSDPTGFSPFPGSVN
360 TLVFKLSSYVDRLRESHGIVPEFINPKYSDETRRSFKKPARIESLMQDIALLFSEDDYRV
420 GGTVFERFSYQPVKNSLEEAAGLVAQGNGAYCAATGEAAFYELQRRRLKAIGLPLFYSSQ
480 PEVTVAKDAFGVRLFPIIVLDTMCASSGSLDDLARVFPTPEKVHIDQHSTLIVEGRVIIE
540 SLELYGALTIRGPTDSMALPHVVRNAVVRNAGWSVHAILSLCAGRDSRLSEVDRIRGFVL
600 KKTAMAVMDCNTKGESEAGAPSGAADPAKL

(*b*)

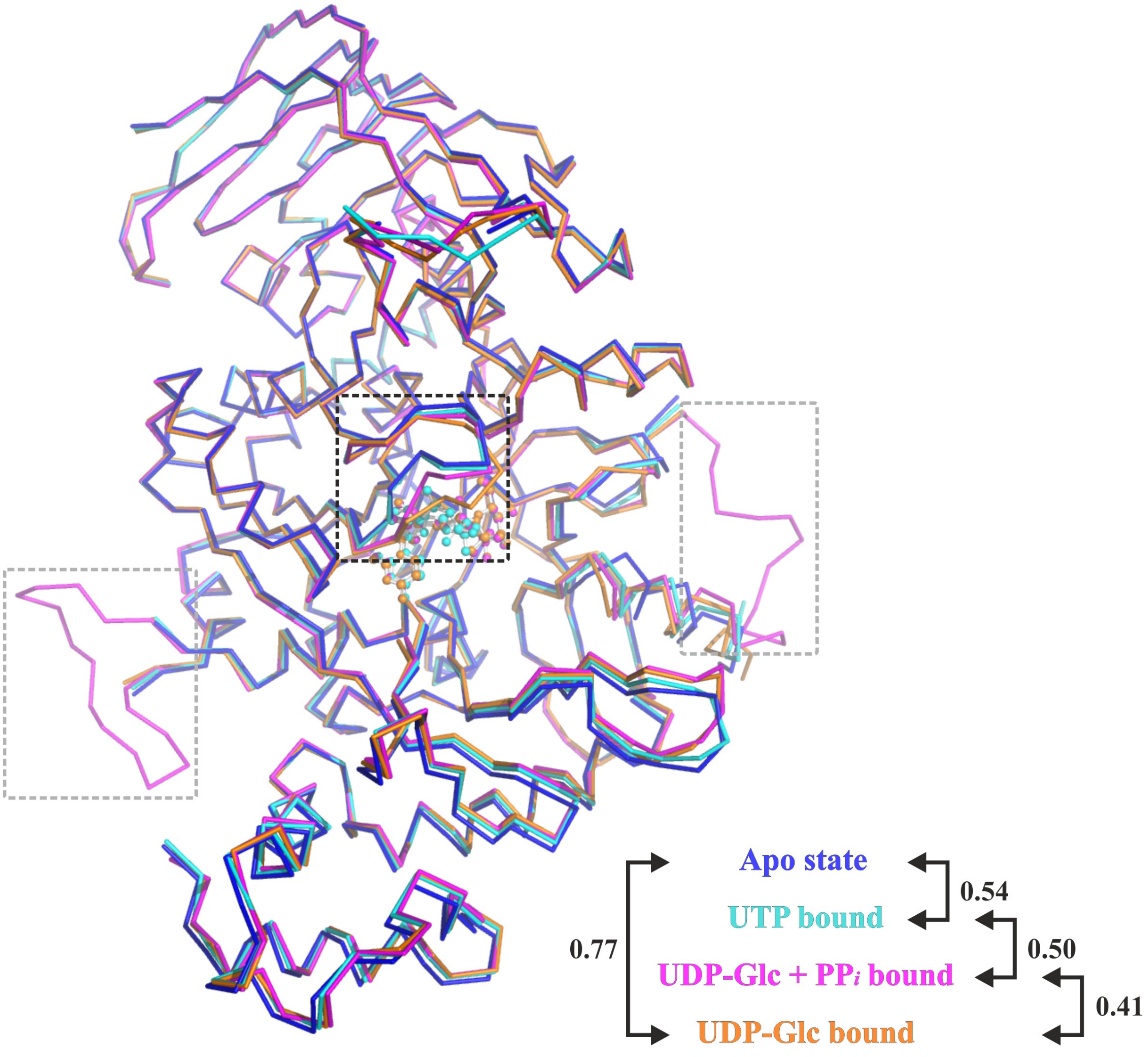
Apo state
UTP bound
UDP-Glc + PP*i* bound
UDP-Glc bound
0.54
0.50
0.41
0.77

(*c*)

Figure S1 The structure and sequence of *Lm*USP. (*a*) The overall structure of *Lm*USP in the substrate-free (apo) state shown in cartoon representation with the N-terminal domain, catalytic M-domain, and C-terminal domain colored in magenta, green, and cyan, respectively. The functional hinge loop 1 and loop 2 at the interface of the N-terminal domain and catalytic M-domain are shown in yellow. The catalytic M-domain includes the NB-loop (red), the SB-loop (blue), and eight parallel β-sheets (orange). (*b*) The sequence of *Lm*USP with secondary structure elements, helices (blue), and β-sheets (orange), indicated above. (*c*) Structural comparison of previously known and new *Lm*USP states, shown in ribbon representation with substrates/products shown in ball-and-stick representation. Structures were overlaid using the SSM superpose function of Coot version 0.9.8.7. Numbers indicate coordinate root mean square deviation (RMSD) (Å) between consecutive states. Previously unresolved regions are boxed in grey, the NB-loop is boxed in black. PDB codes: Apo state 3OGZ, UTP bound state 3OH0, UDP-Glc + PP*i* bound 8TG2, UDP-Glc bound 3OH4.

Figure S2

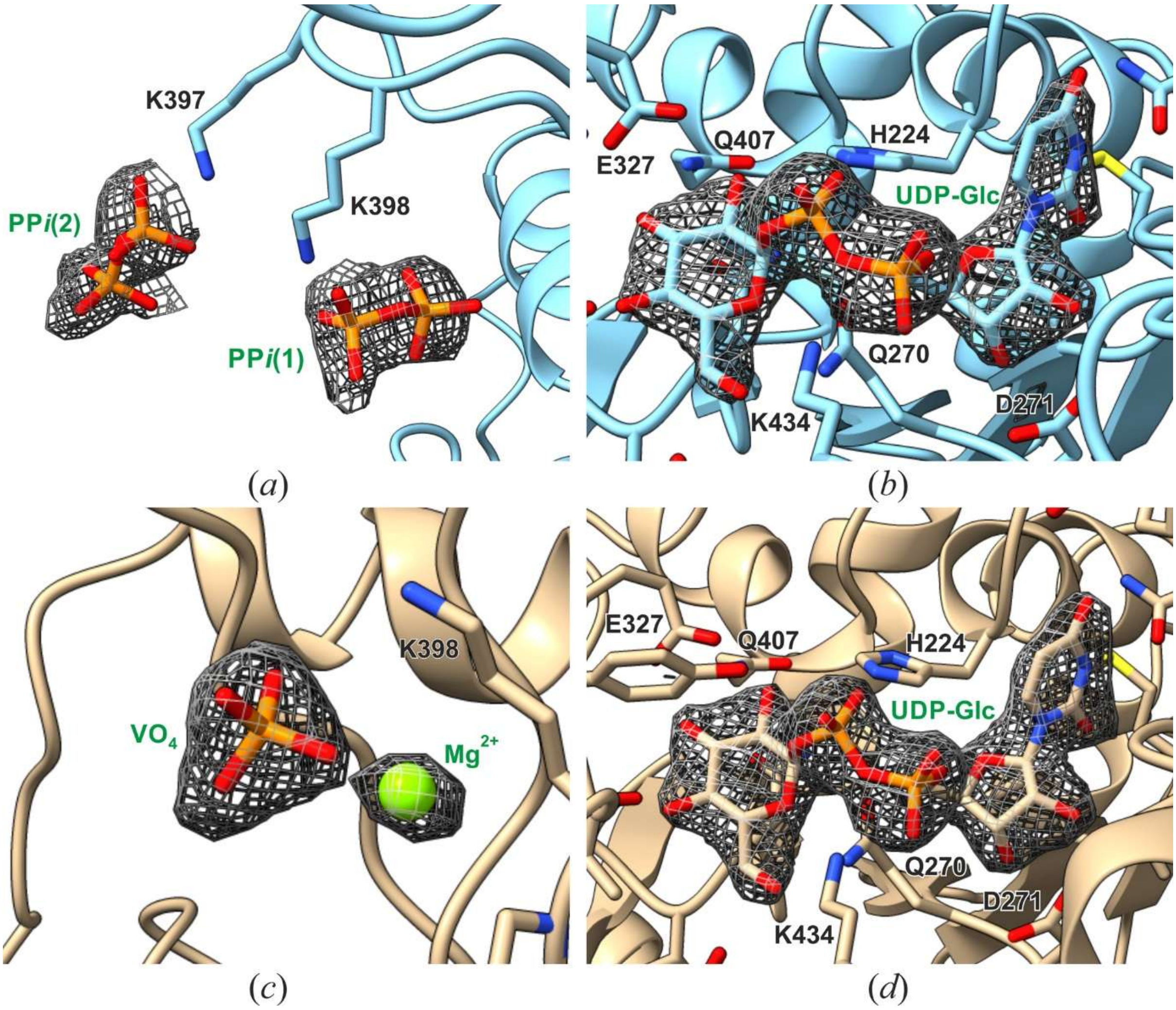

Figure S3 Portions of the composite $2F_{obs}$-F_{calc} electron density omit maps showing (*a*) PP*i* and (*b*) UDP-Glc in the *Lm*USP:UDP-Glc:PP*i* complex (blue); and (*c*) VO_4 with density consistent with a Mg^{2+} ion and (*d*) UDP-Glc in the *Lm*USP:UDP-Glc:VO_4:Mg^{2+} complex (beige). Substrates/products and surrounding active site residues are shown in stick representation, with oxygen, nitrogen, sulfur, and phosphorus in red, blue, yellow, and orange, respectively.

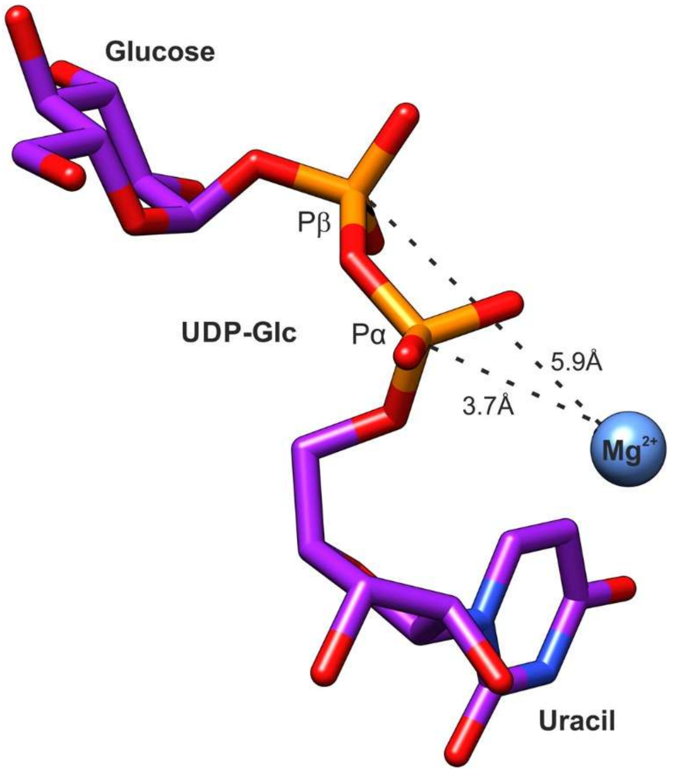

Figure S3 Distances between the Mg^{2+} ion and UDP-Glc:P atoms in *Lm*UGP-UDPGlc-Mg^{2+} complex crystal structure, PDB ID: 4M2A. UDP-Glc is shown in stick, and Mg^{2+} in blue-sphere representations. The distances are shown in black dashed lines.

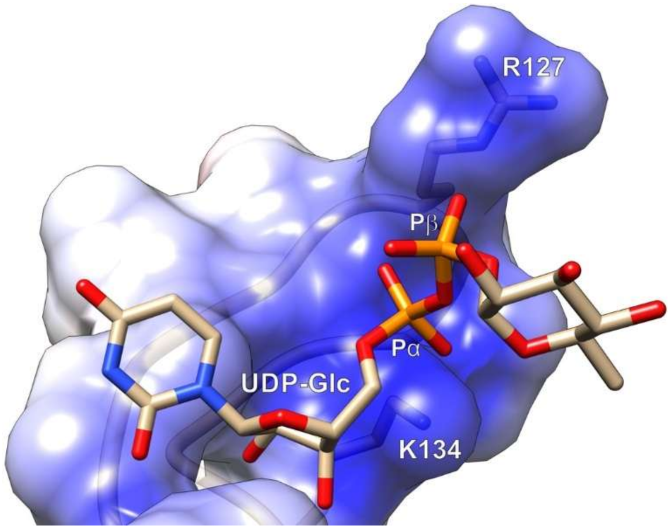

Figure S4 The first coordination site of PP*i* after the catalytic reaction step. The residues R127 and K134 (stick representation) within the active site are likely to coordinate PP*i* immediately after the catalytic reaction step. The molecular surface shows the positive electrostatic potential of the PP*i* coordination site.

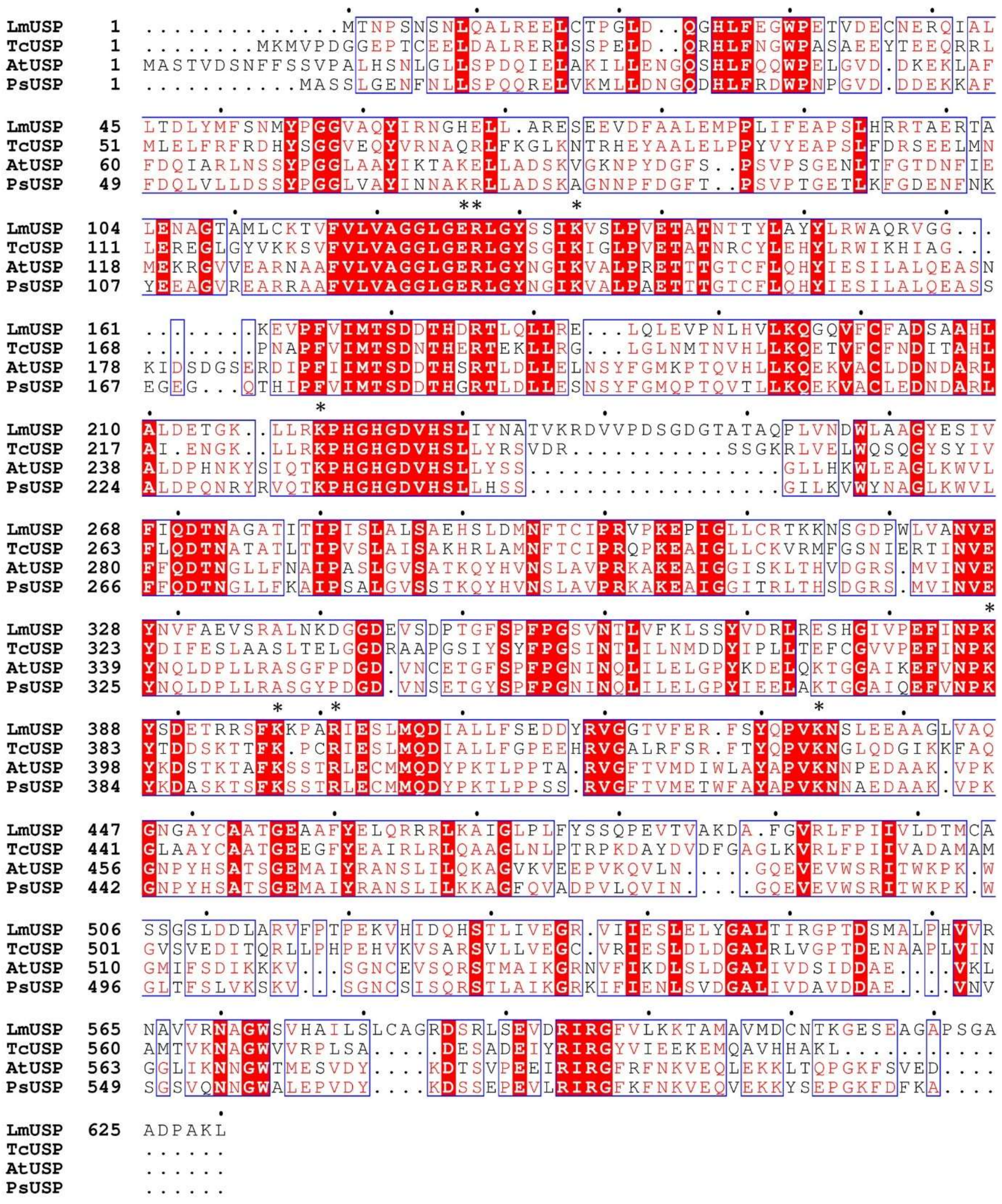

Figure S5 Alignment of UDP-sugar pyrophosphorylase sequences from the protozoan parasites *Leishmania major* (*Lm*USP) and *Trypanosoma cruzi* (*Tc*USP) and from the plants *Arabidopsis thaliana* (*At*USP) and *Pisum sativum* (*Ps*USP). The alignment shows the conservation of residues involved in phosphate binding and the proposed gatekeeper E126 (labeled with asterisks above the alignment). The alignment was generated with the online software Multalin (Corpet 1988 Nucl. Acids Res.).

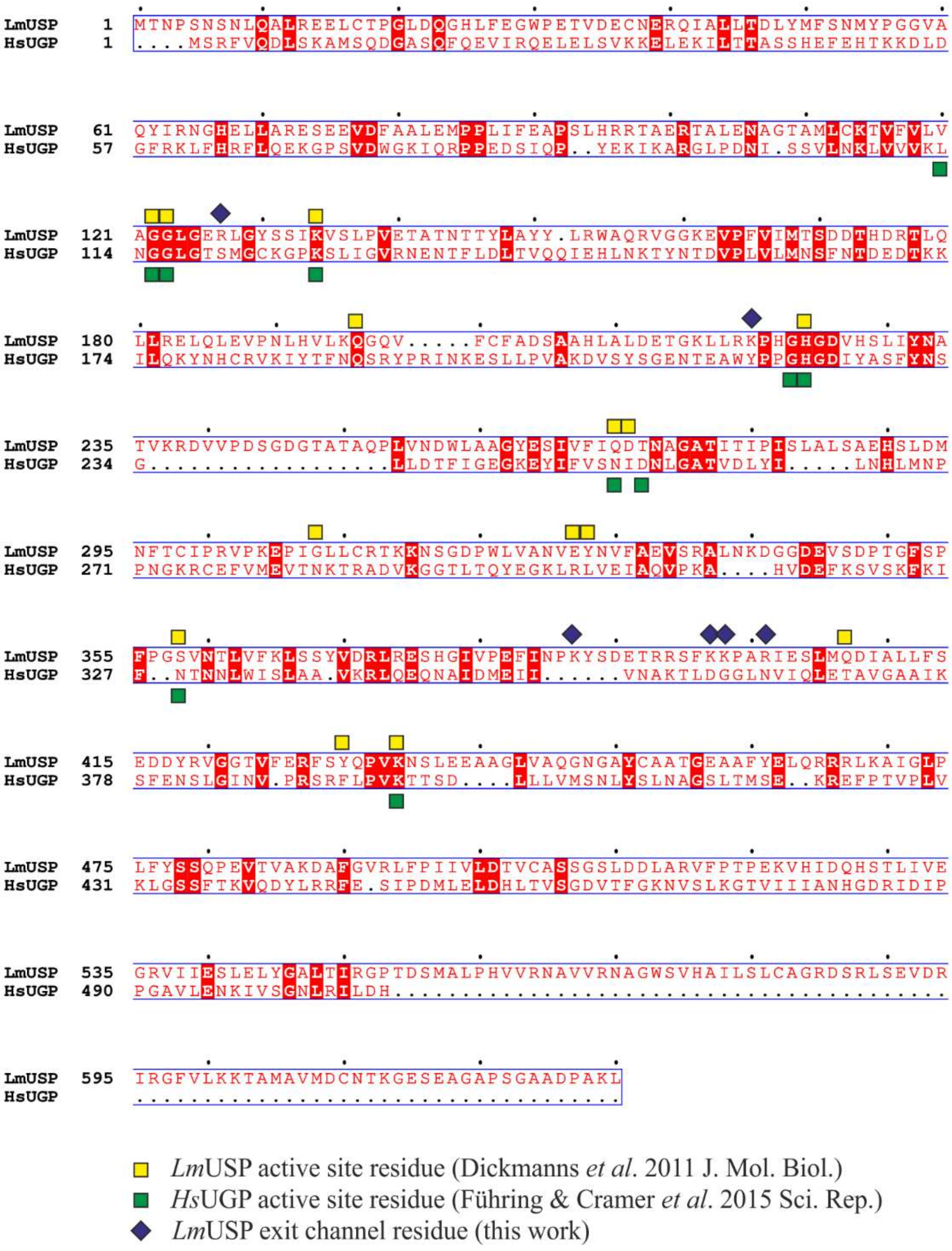

Figure S6 Alignment of *Leishmania major* (*Lm*)USP and *Homo sapiens* (*Hs*)UGP. Active site residues, as well as basic amino acids involved in the *Lm*USP PP*i* exit channel identified in this work, are indicated by symbols. The alignment was generated with the online software Multalin (Corpet 1988 Nucl. Acids Res.).

Table S1 PDB codes and corresponding structural states of USP and UGP structures available in the Protein Data Bank. References: (1) Dickmanns *et al*. 2011 J. Mol. Biol.; (2) Steiner *et al*. 2007 J. Biol. Chem.; (3) Führing *et al.* 2013 ACS Catal; (4) this work.

***Lm*USP PDB Code**	***LmUGP* PDB Code**	**Conformational State**
3OGZ (1)	2OEF (2)	substrate-free (apo) state
3OH0 (1)	4M28 (3)	UTP bound state
3OH1; 3OH2; 3OH3; 3OH4 (1)	2OEG (2)	UDP-sugar bound state
8TG2, 8TGS (4)	4M2A (3)	post-reactive state

Table S2 Coordinate root mean square deviation (RMSD) (Å) between experimentally resolved structural states of *Lm*USP (calculated for protein Cα-atoms).

	**Apo** PDB: 3OGZ	**UTP** PDB: 3OH0	**UDP-Glc:PP*i*** PDB: 8TG2	**UDP-Glc: VO_4:Mg^{2+}** PDB: 8TGS	**UDP-Glc** PDB: 3OH4
**Apo** PDB: 3OGZ	0.00	0.54	0.85	0.84	0.77
**UTP** PDB: 3OH0		0.00	0.50	0.53	0.49
**UDP-Glc:PP*i*** PDB: 8TG2			0.00	0.42	0.41
**UDP-Glc:VO_4:Mg^{2+}** PDB: 8TGS				0.00	0.38
**UDP-Glc** PDB: 3OH4					0.00
